# Nanostructured Tin- and Titanium-Based Chemoresistive Sensors for Detecting Volatile Fingerprints from Colorectal Cancer Biopsies and Cell Lines

**DOI:** 10.3390/ijms27156647

**Published:** 2026-07-25

**Authors:** Michele Astolfi, Martina Masin, Antonio Anfuso, Cesare Malagù, Gabriele Anania, Mascia Benedusi, Giuseppe Valacchi, Giorgio Rispoli

**Affiliations:** 1 Department of Neuroscience and Rehabilitation, University of Ferrara, Via L. Borsari 46, 44121 Ferrara, Italy; stlmhl@unife.it (M.A.); martina.masin@unife.it (M.M.); antonio.anfuso@unife.it (A.A.); mascia.benedusi@unife.it (M.B.); 2 Department of Physics and Earth Sciences, University of Ferrara, Via G. Saragat 1, 44121 Ferrara, Italy; malagu@fe.infn.it; 3 Department of Medical Sciences, University of Ferrara, Via L. Borsari 46, 44121 Ferrara, Italy; ang@unife.it; 4 Department of Environmental and Prevention Sciences, University of Ferrara, C.so Ercole I d’Este 32, 44121 Ferrara, Italy; giuseppe.valacchi@unife.it; 5 Department of Animal Science, Plants for Human Health Institute, North Carolina State University, 600 Laureate Way, Kannapolis, NC 28081, USA; 6 Department of Food and Nutrition, Kyung Hee University, Seoul 02447, Republic of Korea

**Keywords:** gas sensors, nanostructures, potential barriers, cancer, VOCs, chemoresistivity, sensitivity, sensor device, fingerprint, colorectal cancer

## Abstract

Colorectal cancer represents a global health burden, being the third most frequently diagnosed cancer worldwide, accounting for about 1.9 million new cases annually, and the second leading cause of cancer-related deaths. This highlights the need for innovative and effective methods for cancer detection, improving current diagnostic approaches. In this feasibility study, two nanostructured chemoresistive gas sensors based on tin oxide–titanium oxide composites were selected to detect the metabolic patterns associated with human healthy and colorectal cancer tissues, collected from 26 patients. To further validate the biopsy-derived results, the sensors were tested on two colorectal cancer-derived cell lines, namely Caco-2 and RKO. Both sensors were able to discriminate between healthy and tumor samples by comparing the mean sensor responses, using the paired *t*-test (*p*-value = 0.00018), and by using an LDA, achieving a cross-validated accuracy of 0.73. Moreover, the ROC analysis performed on the data projected along the discriminant direction led to an AUC of 0.83. Finally, the sensors exhibited distinct response profiles to Caco-2 and RKO exhalations, as expected given their different biological features. As a proof-of-feasibility study, these findings demonstrate that these sensors could capture a characteristic volatile fingerprint distinguishing the samples, although they cannot identify the specific cancer metabolites.

## 1. Introduction

Cancer remains a leading cause of mortality worldwide, with nearly 20 million new cancer cases diagnosed in 2022 [1]. Among these, colorectal cancer (CRC) accounted for approximately 1.93 million new cases globally (9.6% of all cancer diagnoses), ranking as the third most commonly diagnosed cancer and the second leading cause of cancer-related death, with more than 900,000 deaths reported worldwide [2,3,4]. Cancer screening programs can reduce both cancer incidence and mortality by detecting and removing precancerous lesions, thereby preventing progression to invasive disease and enabling treatment at earlier stages [5]. Indeed, early diagnosis and treatment (both surgical and pharmacological) of many types of cancer, including CRC, are associated with reduced mortality and improved long-term survival, particularly when the disease is detected at an early stage, while it remains fully localized and can be completely removed through surgery [5,6,7,8]. Current clinical screening and prevention protocols for CRC rely on the Fecal Immunochemical Test (FIT) [9], high sensitivity guaiac Fecal Occult Blood Test (HSgFOBT), and colonoscopy, among other approved clinical strategies [10]. Colonoscopy is judged as the gold standard for CRC detection, allowing a direct observation of the lesions and allowing a concurrent polypectomy (if needed). Nevertheless, despite its high diagnostic accuracy, colonoscopy is associated with a certain risk of failure, as it is primarily a visual examination and may therefore miss some lesions, particularly in the presence of suboptimal bowel preparation or rapidly progressing tumors [11,12]. Furthermore, colonoscopy is an invasive procedure that requires extensive bowel preparation and is associated with rare but potentially serious complications, such as bleeding and bowel perforation, making the procedure not always acceptable and applicable to some patients [13]. Given that, the call to the scientific community concerns the development of innovative and non-invasive technologies for CRC detection, preferably at a preliminary stage, with the aim of improving currently available screening methods and protocols, thereby reducing CRC-associated mortality. A field of major interest today concerns the study and detection of volatile organic compounds (VOCs) as potential tumor biomarkers, since VOCs are a broad class of low-molecular-weight carbon-based compounds characterized by a high vapor pressure, which allows them to readily evaporate at ambient temperature [14,15,16]. It has been demonstrated in the literature that VOCs emitted by cells directly reflect their metabolic activity, which is profoundly altered in cancer cells as a consequence of metabolic reprogramming, enhanced proliferation, oxidative stress, and dysregulated lipid and amino acid metabolism [17,18,19,20]. These alterations lead to the production of distinct VOC patterns that can be exploited as potential biomarkers for cancer detection, as indeed found for CRC [21,22]. VOC-based gas sensing is conceptually connected to the broader field of liquid biopsy, which also represents a minimally invasive approach for cancer detection, although it relies on different types of biological information. On the one hand, VOC sensing analyzes volatile metabolic patterns in breath or the headspace of biological fluids, offering a rapid, low-cost, and potentially non-invasive approach to cancer screening. On the other hand, liquid biopsy biosensors detect tumor-associated biomarkers in blood or other body fluids, including circulating tumor DNA, extracellular vesicles, circulating tumor cells, proteins, and metabolites, providing more molecularly specific information for diagnosis, prognosis, treatment selection, and disease monitoring [23,24]; in any case, VOC sensing remains attractive for simple front-line screening. Based on their expertise, the research group at the “Laboratorio Sensori” of the University of Ferrara, having already gained considerable experience in sensor systems for the detection of tumor-related VOCs from various biological samples such as blood, cell cultures, and stools [25,26,27,28], has developed a new sensor device aimed at identifying VOCs emitted by tumor cells directly from biopsy samples collected from tumors in the operating room. In this feasibility study, two sensors based on tin, titanium, and niobium oxides were selected and installed in the aforementioned device to analyze volatile emissions from healthy and diseased human tissue samples, collected from 26 patients who underwent colorectal cancer surgery at Sant’Anna Hospital in Cona (Ferrara), with the aim of distinguishing the two metabolic patterns. As an additional assessment of the sensors’ ability to identify tumor samples, the volatile emissions of two colorectal cancer-related cell lines, RKO and Caco-2, were analyzed. As mentioned previously, this work was conceived as a preliminary feasibility study rather than an attempt to validate a clinical screening tool, as its aim was to detect tumor-specific fingerprints using this type of sensor and to pave the way for future research.

## 2. Results

The results of this study are organized into distinct sections, addressing the different experimental models investigated. In particular, measurements performed on biopsy samples and on the two immortalized cell lines are presented separately to provide a clearer interpretation of the sensor responses. The Section 2 begins with the identification of the optimal operating temperature of the sensors, which represents a crucial preliminary step for the subsequent analyses.

### 2.1. Sensors’ Operating Temperature Selection

As a preliminary step, the optimal operating temperature of the sensors employed in this study was evaluated by exposing both sensors to three DMEM samples and three water–ethanol mixture samples at each tested temperature. The temperature range investigated spanned from 300 to 600 °C, with increments of 50 °C.

The reference conditions used to determine the optimal operating temperature were the same as those employed during the device’s field operation: environmental air, filtered and stabilized using an activated carbon filter and a 0.2 µm filter, at an ambient temperature ranging from 25 to 27 °C and a relative humidity between 20% and 30%.

**Figure 1:**
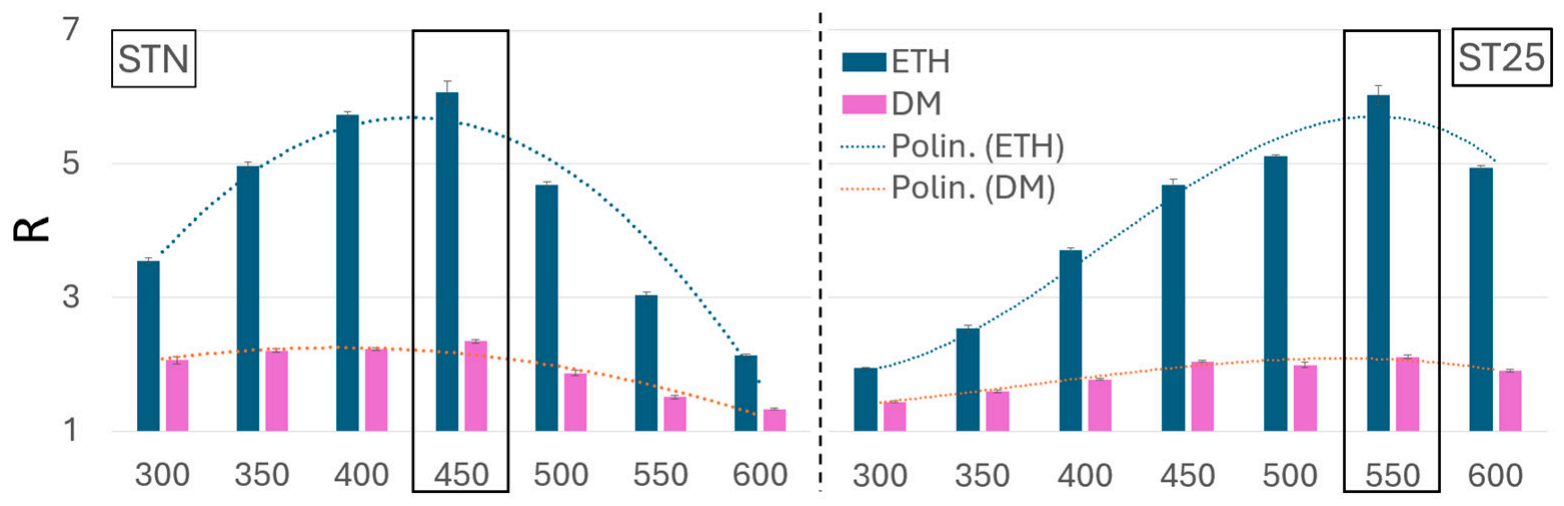
Best working temperature selection. Average sensor responses (*n* = 3) to DMEM (DM, purple bars) and ethanol–water solution (ETH, blue bars) measured at operating temperatures ranging from 300 °C to 600 °C, in 50 °C increments. The (**left**) and (**right**) panels correspond to the STN and ST25 sensors, respectively. The standard error bars turned out to be almost negligible; the black rectangle indicates the optimal working temperature.

The aim of this procedure was to identify the operating temperature at which the sensors exhibited the highest sensitivity to ethanol, selected as a volatile organic compound (VOC) for sensor calibration, while maintaining a stable response to DMEM, which was used as the culture medium for the preparation of both cellular and biopsy samples. It is worth noting that these optimal operating temperatures were selected by considering the overall sensor performance, including response magnitude, signal stability, and dynamic behavior (see Section 4). The selected temperatures, 550 °C for ST25 and 450 °C for STN (Figure 1) provided the highest response to the ethanol-water solution and the most stable response to DMEM. Moreover, compared with the immediately lower temperatures (500 °C for ST25 and 400 °C for STN), they exhibited faster recovery times, while the response times remained comparable. Conversely, further increasing the operating temperature (600 °C for ST25 and 500 °C for STN) resulted in higher baseline and signal fluctuations, lower sensor responses, and slightly longer response and recovery times.

### 2.2. Biopsy Analysis

The focus of this study is the analysis of volatile gaseous emissions from human colorectal biopsy samples, specifically healthy mucosal tissue (collected as far as possible from the tumor site) and tumoral tissue (collected directly from the neoplastic lesion). Both tissue types were obtained from surgical specimens following colorectal tumor resection.

**Figure 2:**
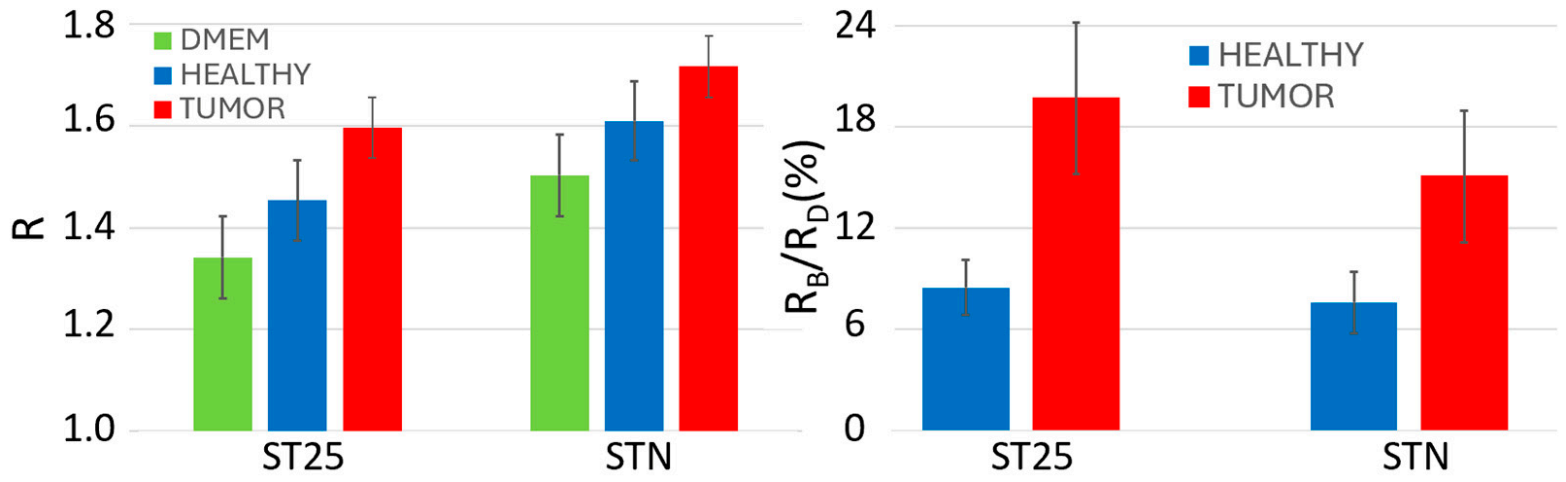
Average sensor responses. (**Left Panel**): Average sensor responses (*n* = 26) to DMEM (green), healthy (blue), and tumoral samples (red), the numeric values are reported in Table 1; (**Right Panel**): Average sensor responses (*n* = 26) with respect to DMEM expressed as percentage, where R_B_ and R_D_ are the responses to biopsy and DMEM samples, respectively. The error bars represent the standard errors.

The average responses (*n* = 26) and the related standard errors of the ST25 and STN sensors to healthy and tumoral tissues were computed for a total of 26 patients (Figure 2, Table 1). DMEM (*n* = 52) was used as a standard and constant reference sample for system calibration, being measured before and after the biological samples (Figure 2, left panel). In order to make the responses independent of environmental and instrumental variations, the average responses of the two sensors were expressed as a relative value to the DMEM ones; in Figure 2 right panel and in Table 2, they were reported as percentages. A clear difference emerged between the mean responses of healthy and tumor samples for both sensors investigated.

To assess whether the differences shown in Figure 2 (right panel) were statistically significant, a paired *t*-test was performed on the log-transformed values of the healthy and tumor samples from each patient. This analysis revealed a statistically significant difference between the mean values of the paired samples (*t* = 6.2, *p* = 0.00018 < 0.05), and the estimated mean difference log (tumor)—log (healthy) was supported by a 95% confidence interval ranging from 0.04 to 0.09. To assess the magnitude of the observed difference, Cohen’s *d* was calculated, yielding an effect size of 1.22, which generally indicates a significant effect.

In order to explore the correlations among sensor responses and sample weight (with an average of 0.29 ± 0.18 g; n = 26) a correlation matrix, with a relative heatmap, was computed on the data, under the assumption that larger samples are expected to emit a greater amount of volatile compounds.

**Figure 3:**
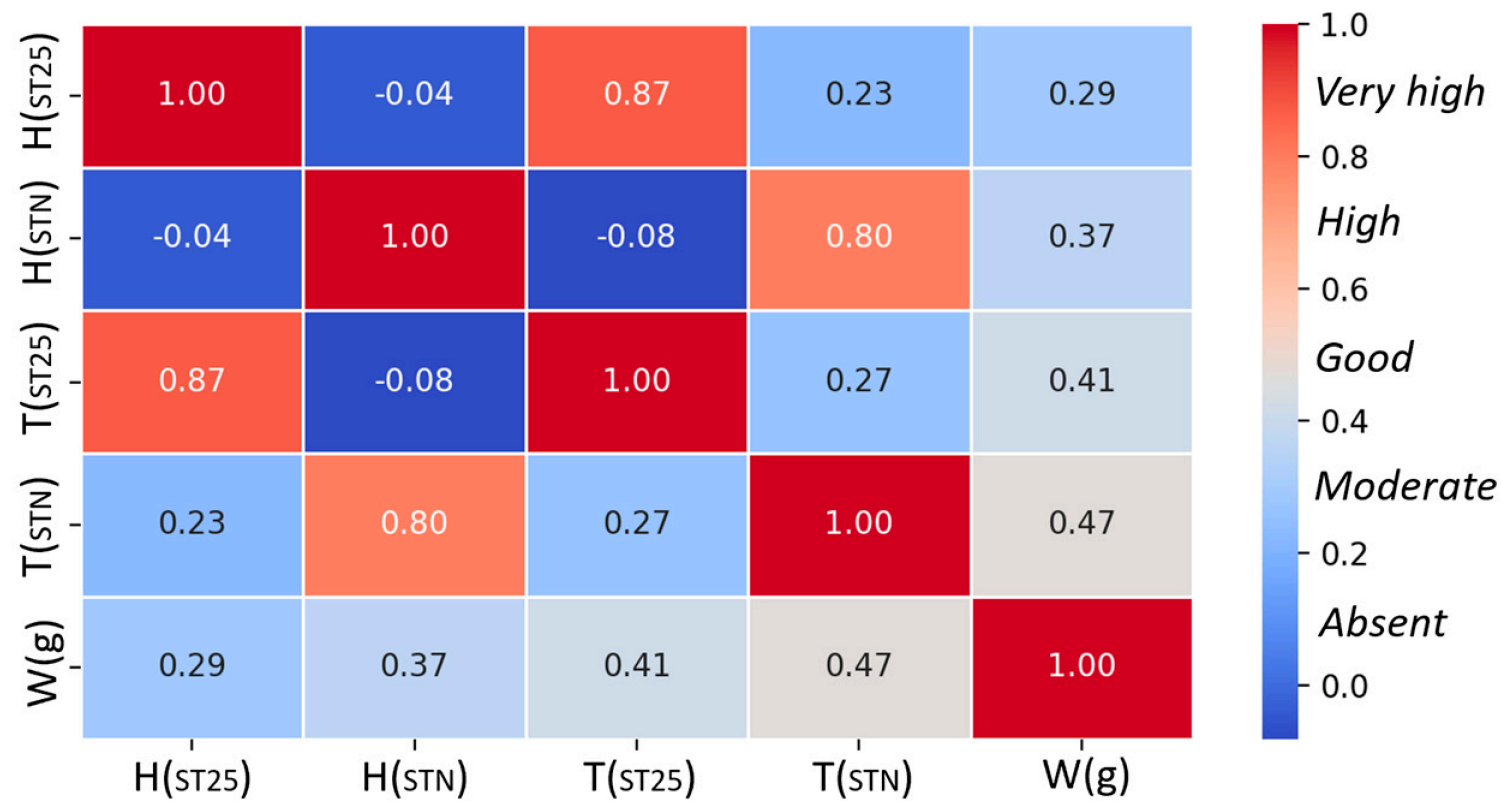
Correlation matrix between the sensor responses (H = healthy, T = tumor) and the biopsy sample weight W (g).

The matrix (Figure 3) shows an absence of or a very weak correlation between the two sensors (ST25 and STN) to healthy (r = −0.04; *p*-value = 0.84) and tumor (r = 0.27; *p*-value = 0.19) samples; a moderate but not statistically significant correlation between the responses of the two sensors to healthy tissues and the sample weights (0.29 and 0.37 for ST25 and STN; *p*-values of 0.15 and 0.065, both >0.05); and finally, a good and statistically significant correlation between the responses of the two sensors to tumor samples and the sample weight (0.41 and 0.47 for ST25 and STN; *p*-values of 0.036 and 0.016, both < 0.05).

**Figure 4:**
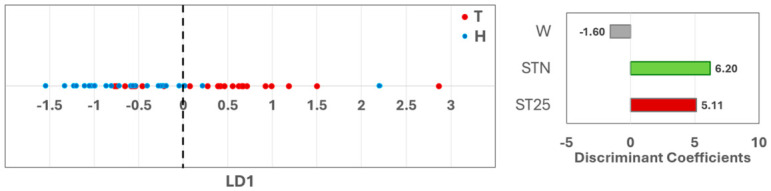
LDA. LDA data plot on LD1 direction (**left**); bar plot of discriminant coefficients (**right**); grey box refers to sample weight, green one to STN, and red one to ST25.

Healthy and tumoral samples were classified by Linear Discriminant Analysis (LDA) using the DMEM-normalized responses of the two sensors and the sample weight as input features [29]. Because only two classes were considered (healthy and tumor), a single linear discriminant (LD1; 100% of the discriminant variance) was obtained. The samples were projected onto LD1, which maximizes the separation between the healthy and tumoral groups (Figure 4). In particular, the discriminant coefficients were 5.1, 6.2, and −1.6 for the ST25 sensor, the STN sensor, and the sample weight, respectively (Figure 4, right panel). The class centroids were located at −0.53 and 0.53 for the healthy and tumor classes, respectively.

The performance of the LDA classifier was assessed using accuracy, average cross-validated accuracy, precision, recall, and F1-score (Table 3). The model achieved an accuracy of 77% and an average accuracy of 73%, while precision, recall, and F1-score were computed for each class and reported in Table 3. Furthermore, the confusion matrix was reported in Table 4.

The LDA model showed a statistically significant discriminant function (Wilks’ Lambda = 0.76, F (3, 48) = 4.9, *p* = 0.0045), indicating that the combination of the selected variables provided significant discrimination between the healthy and tumor classes.

**Figure 5:**
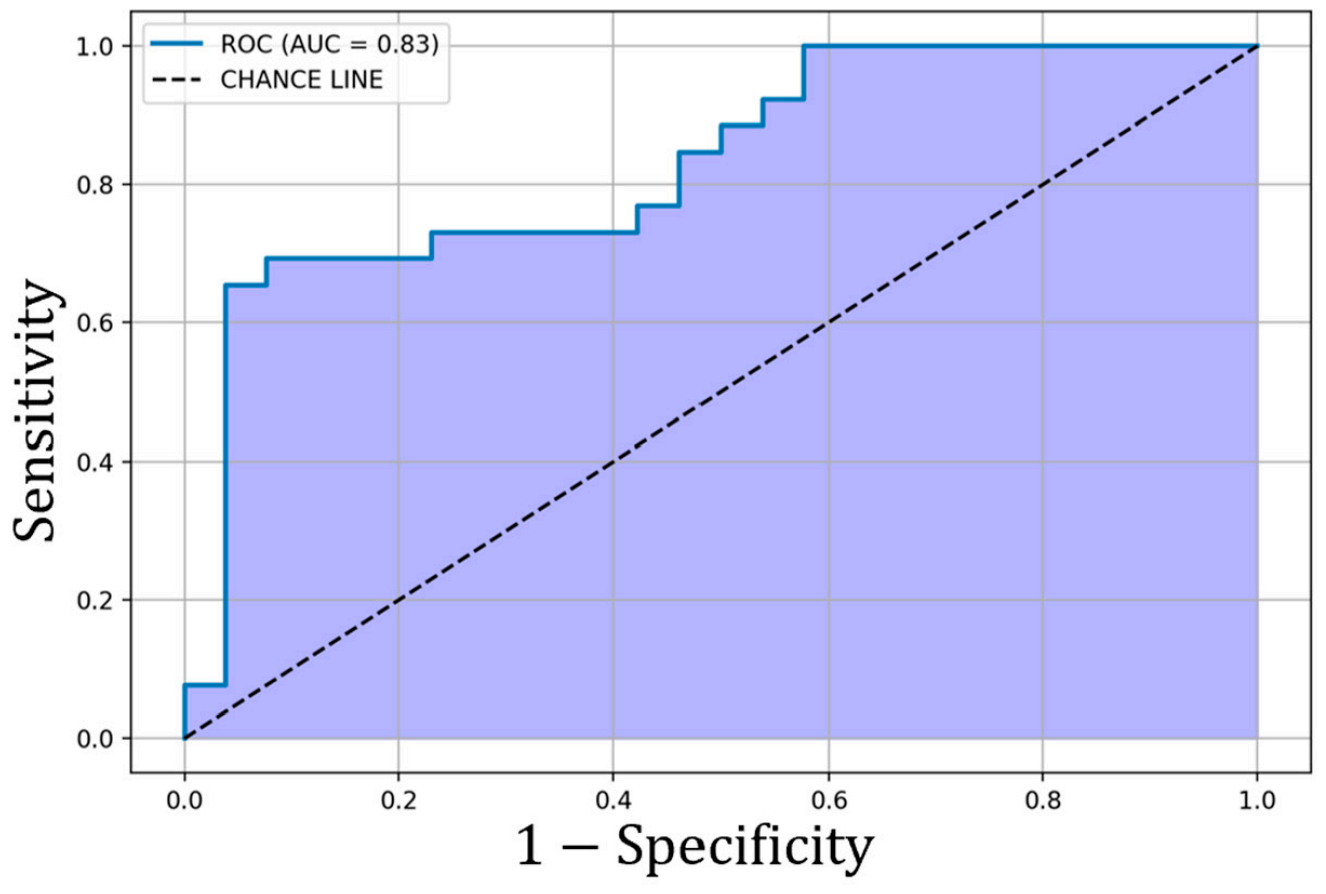
Receiver operating characteristic (ROC) curve based on the data projected onto the LD1 axis. The ROC curve is shown as the blue solid line, and the shaded blue area represents the area under the curve (AUC). The black dashed diagonal line indicates the performance expected by chance.

The ROC curve analysis resulted in an AUC of 0.83 (Figure 5), indicating that the LDA model had good discriminatory ability. An AUC above 0.80 is generally considered to reflect good classification performance, suggesting that the model effectively distinguished between healthy and tumor samples [30].

### 2.3. Immortalized Cell Culture Analysis

Two human colorectal cancer-derived immortalized cell lines were investigated, namely RKO [31], selected to represent an aggressive colorectal cancer phenotype, and Caco-2 [32]. Although both are human epithelial cell lines originating from CRC, they differ markedly in their genetic background, differentiation status, morphology, and experimental applications. Caco-2 spontaneously differentiates into enterocyte-like cells that resemble those of the small intestine; consequently, Caco-2 cells are generally used to investigate intestinal absorption, drug permeability, epithelial barrier function, and host–microbiome interactions [33]. In contrast, RKO cells are a poorly differentiated CRC cell line, commonly regarded as a model of a distinct molecular subtype of CRC (see Section 3).

**Figure 6:**
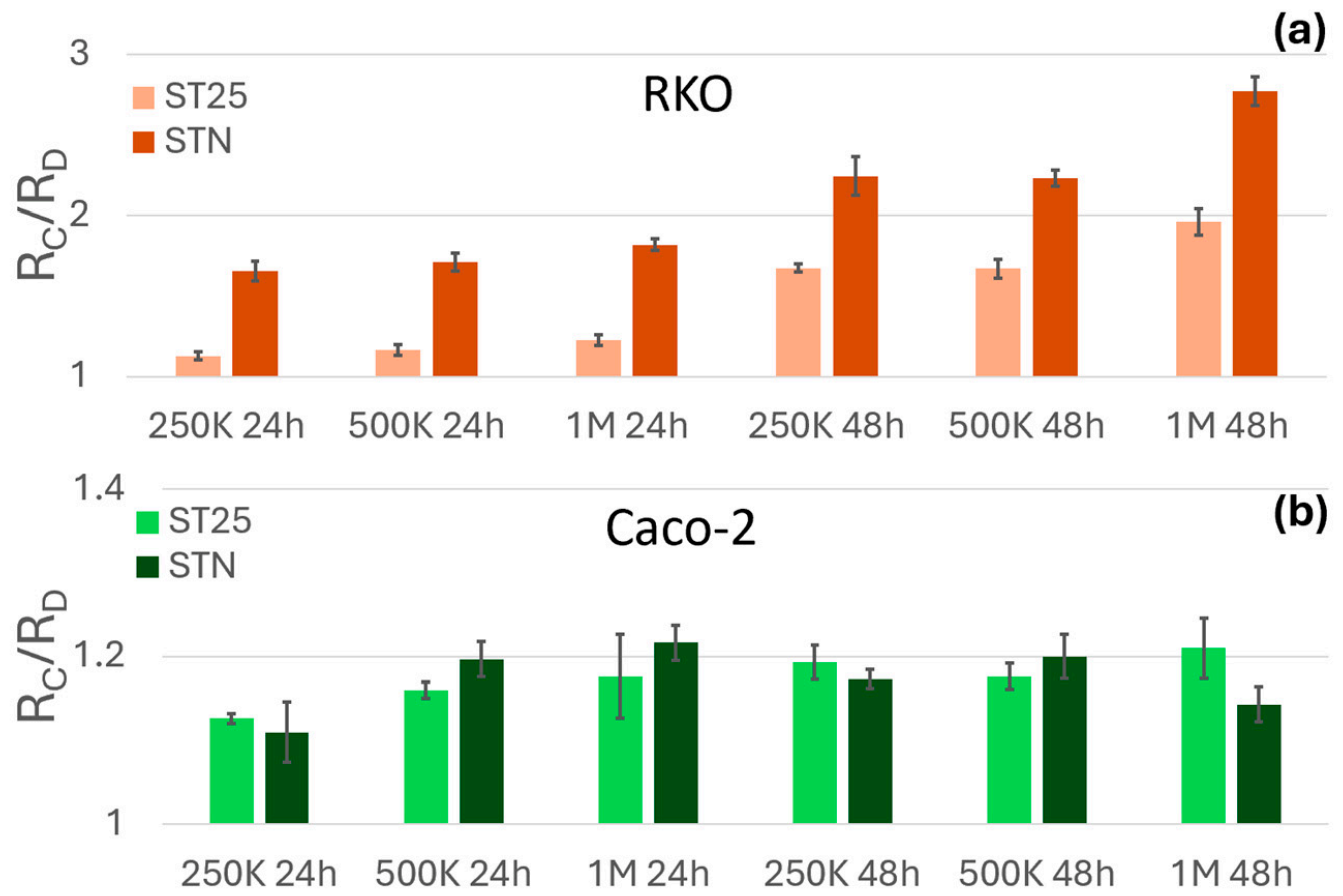
Average DMEM-normalized sensor responses (*n* = 3 replicates) to (**a**) RKO and (**b**) Caco-2 cell lines seeded at different initial concentrations (250 K, 500 K, 1 M) and measured after two different incubation times (24 and 48 h).

**Figure 7:**
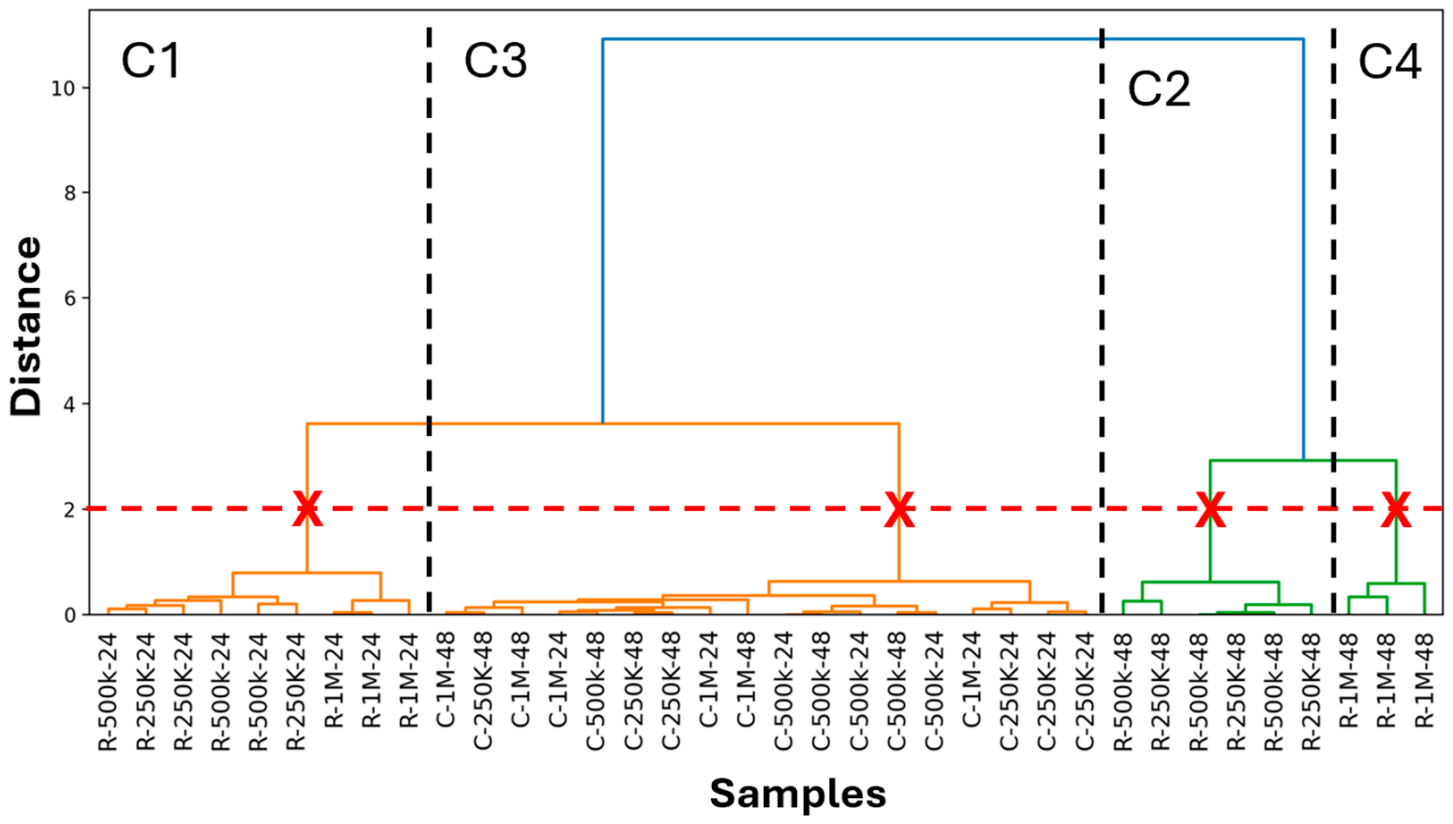
Hierarchical agglomerative clustering dendrogram. C1, C2, C3, and C4 are the clusters detected by cutting the dendrogram at height *d* = 2.0, as justified by Silhouette score (see Figure 8 and Table 4). This clustering analysis provides a visual representation of the similarity between samples and suggests a tendency toward separation between the analyzed groups.

The histograms of the average DMEM-normalized responses of the two sensors to RKO (Figure 6a) and Caco-2 (Figure 6b) and their corresponding standard errors (see Materials and Methods) highlighted a clear difference between the two cell lines. Moreover, only the RKO cell line showed a discernible pattern of increased volatile emissions as a function of both incubation time and initial cell plating concentration. The cell samples were subsequently classified using hierarchical agglomerative clustering with the “ward” linkage method and “euclidean” distance; the resulting dendrogram is shown in Figure 7. The dendrogram was cut at a height *d* = 2.0, identifying four distinct clusters containing 9, 18, 6, and 3 samples (Table 4), respectively. This clustering method allows the identification of natural groups of samples considering their multivariate similarity, without the need for an a priori chosen number of groups; the “ward” method was adopted because it usually generates compact and relatively homogeneous clusters by minimizing within-cluster variance. However, it must be clarified that the observed clustering represents only a pattern of sample similarity and, by itself, suggests a possible separation between groups rather than a confirmed biological classification.

**Figure 8:**
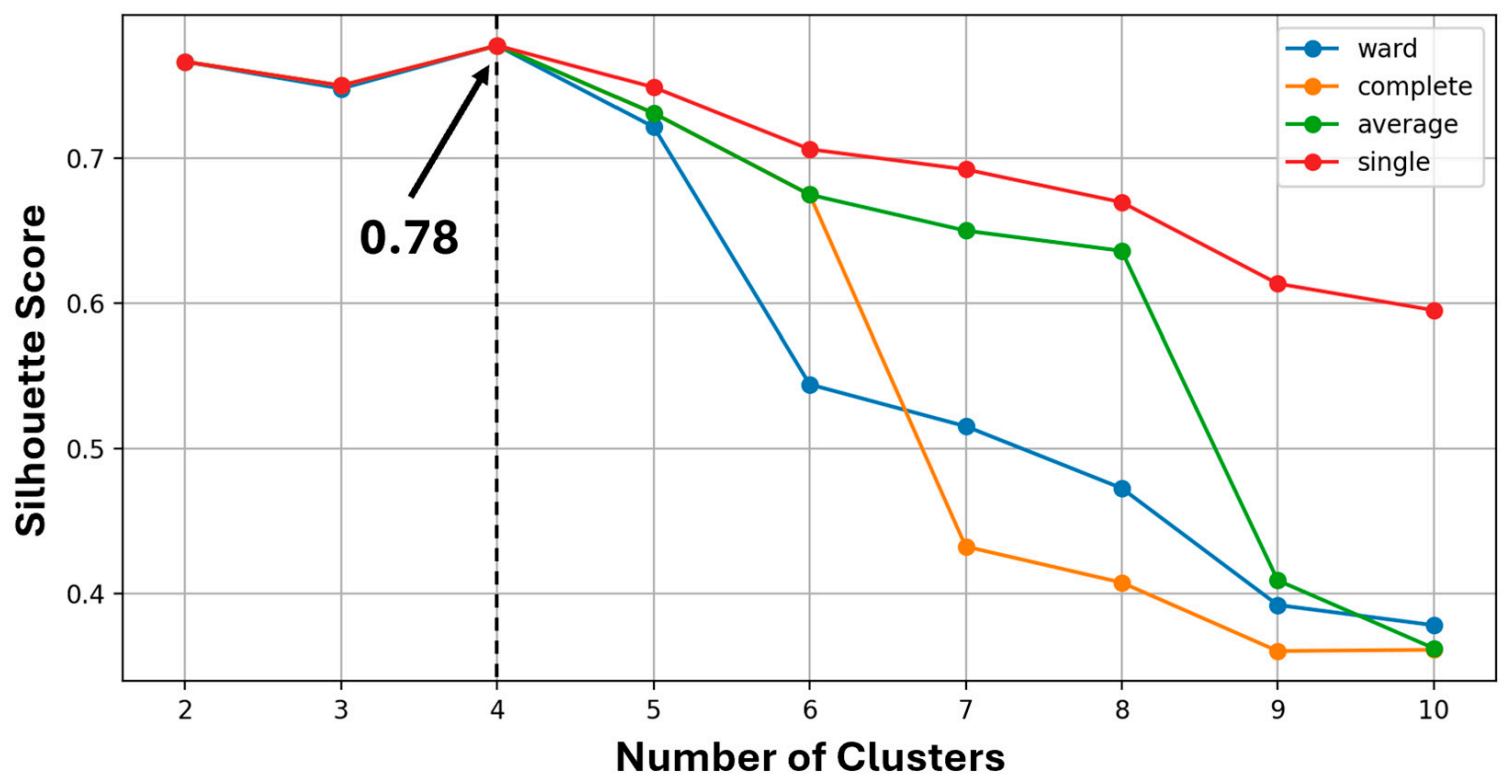
Plot of silhouette scores across different linkage methods and numbers of clusters, ranging from 2 to 10; black arrow indicates the maximal silhouette score corresponding to optimal number of cluster indicated by the dotted line.

To determine the most appropriate number of clusters and identify the optimal point at which to cut the dendrogram for the most meaningful classification of the data, the average Silhouette score [34] was computed (Figure 8) for different linkage methods and numbers of clusters (ranging from 2 to 10). The silhouette score is the most widely used and reliable technique for quantitatively evaluating whether the data are well clustered by returning a value between −1 and +1; the closer the value is to +1, the better the clustering result. The best solution was obtained for N = 4 clusters for all the linkage methods (Figure 8 and Table 5), which yielded the highest score of 0.78. However, the highest silhouette score was used to identify the mathematically optimal clustering solution and number of clusters, although this metric alone does not establish the biological significance of the identified clusters, which should be interpreted in conjunction with the experimental results.

## 3. Discussion

The results demonstrate that both ST-selected sensors successfully discriminated tumor samples from healthy ones, suggesting that cancer-related cellular alterations produce a detectable volatile chemical signature [35,36]. Indeed, as reported in the literature, cancer cells emit different volatile compounds compared to healthy cells due to cellular alterations occurring only in tumor cells, such as increased oxidative stress, membrane lipid peroxidation, and changes in cellular metabolic pathways, favoring glycolysis as the primary source of energy [17,18,20]. These processes can generate specific VOCs that are released into the surrounding environment and detected by gas sensors [37,38].

Indeed, most volatile organic compounds (VOCs) associated with tumor metabolism behave as reducing species when interacting with n-type MOX gas sensors, since they can be subjected to surface oxidation with adsorbed oxygen ions. This mechanism leads to electron return to the conduction band and a consequent increase in the sensor conductance; this mechanism is well documented for typical cancer-related reducing gases such as aldehydes, ketones, and so on. This finding supports the observation that both ST-based sensors employed in this study, although uncorrelated, produced significantly higher responses in tumoral samples than in healthy tissues.

To further validate the biopsy results, the potential of the sensors was assessed by using two CRC cell lines, Caco-2 and RKO, which are widely employed in gastrointestinal research. The sensor responses were markedly different between Caco-2 and RKO cells: Caco-2 cells did not induce a progressive change in sensor responses as a function of initial cell concentration or incubation time, whereas responses to RKO cells were significantly higher and showed a progressive increase with both initial concentration and incubation time, as demonstrated by the mean analysis and hierarchical clustering. These findings are readily explainable by considering the substantial differences between the two cell lines investigated. Caco-2 cells derived from a colorectal adenocarcinoma but spontaneously differentiate upon confluence into enterocyte-like cells, forming polarized epithelial monolayers with tight junctions and well-developed microvilli. Because they display many features of differentiated intestinal epithelium rather than aggressive CRC, they are commonly used to study intestinal absorption, drug permeability, epithelial barrier function, and host–microbiome interactions [33,39]; therefore, Caco-2 cells were considered here as a model of a low-grade CRC tumor. In contrast, RKO cells are a poorly differentiated CRC cell line representing a distinct molecular subtype of colorectal cancer. Unlike Caco-2 cells, which are microsatellite stable, RKO cells show high microsatellite instability, a condition in which short, repetitive DNA sequences called microsatellites become unusually prone to gaining or losing repeats because the cell’s DNA repair system is not working properly. Moreover, RKO cells carry the B-Raf V600E mutation [40,41] where valine is replaced by glutamic acid at position 600: this mutation keeps B-Raf signaling permanently active, promoting cell growth even without external growth signals; therefore, RKO cells were used here because they mimic a high-grade and aggressive tumor tissue. However, the absence of genuinely non-malignant human colonic epithelial control, such as FHC cells or normal colonic organoids, limits the extent to which the observed VOC signatures can be conclusively attributed specifically to malignant cells. Addressing this limitation will be an important priority for future validation studies.

While the results are highly encouraging and provide a strong rationale for future investigations, it is important to acknowledge the limitations and potential risks of bias associated with the present study. First of all, the surgically collected samples, especially the tumor ones, may be extremely small (in this study, the smallest one was 0.08 g), because when the tumor mass is very small, only a limited amount of tissue can be collected from the surgical specimen by surgeons. This is due to the need to preserve the specimen for pathological examination, which is required to characterize the neoplasm (e.g., determine its stage, grade, and other pathological features); however, paired healthy and CRC tissues from the same patient were always analyzed using identical sample weights as detailed in Section 4.2. Therefore, the VOC emission is expected to be reduced due to the small number of cells present in the sample; this is indeed supported by the correlation matrix, which highlights a moderate statistically significant correlation between sensor responses and sample size, particularly for the STN sensor. Nevertheless, equal gross weight does not necessarily indicate equal cellularity, because colorectal carcinomas and normal colonic mucosa have fundamentally different histological architectures, particularly in the relative proportions of epithelial cells, stroma, mucus, and necrotic tissue. Without normalization to DNA or total protein content, the observed differences in VOC emissions may partly reflect differences in viable cell density or volume rather than isolated metabolic alterations.

Another noteworthy limitation was bacterial contamination, which resulted in the exclusion of six samples after microscopic examination, reducing the number of samples available for evaluation. The difference in VOC emissions between CRC tumor tissue and matched healthy tissue from the same patient was consistently observed across all biopsies. Thus, any residual adherent bacterial biofilm that was not removed during washing would be expected to be present at comparable levels in both samples from the same patient, in agreement with previous reports [42], given that the two specimens were collected only a few centimeters apart. However, other studies [43] suggest that the colorectal tumor microenvironment actively recruits and supports a distinct microbiota, including *Fusobacterium nucleatum*, thereby creating a localized microbial niche that differs markedly from the adjacent normal mucosa. Because this bacterium can become incorporated into mucosa-associated biofilms, it may not be completely removed by routine sample-washing procedures. Consequently, the possibility that the sensors detect VOCs produced by the tumor-associated microbiome, rather than exclusively by the cancer cells themselves, remains a plausible alternative explanation. Nevertheless, this hypothesis would not account for the differences in VOC emissions detected between the Caco-2 and RKO cell lines, both of which were free of bacterial contamination.

Since measurements were conducted using a portable device and filtered environmental air, employed both as a sensor stabilizer and VOC carrier, the main potential source of bias was the variability in chemoresistive MOX sensor performance caused by environmental fluctuations, which were nevertheless kept within acceptable limits. To mitigate this bias, the selected sensor materials were chosen because they are relatively insensitive to fluctuations in environmental conditions [44] and all measurements were systematically normalized against DMEM control samples, which were consistently measured at the beginning and at the end of each day of analysis. Finally, the use of sensors for VOC detection remains a highly attractive and promising field of research, with considerable potential for future clinical applications. However, to fully exploit their diagnostic potential, sensor-based approaches should be integrated with complementary analytical techniques, such as liquid biopsy, GC-MS, PTR-MS, SIFT-MS, and other analytical chemistry methods capable of identifying the VOC classes underlying the sensor response (including aldehydes, ketones, alcohols, and hydrocarbons); this multimodal integration could provide more complete tumor-specific information for precision oncology, thereby enhancing diagnostic sensitivity, improving biological interpretation, and supporting clinical decision-making.

## 4. Materials and Methods

### 4.1. Nanostructured Chemoresistive Sensors and Their Working Principle

**Figure 9:**
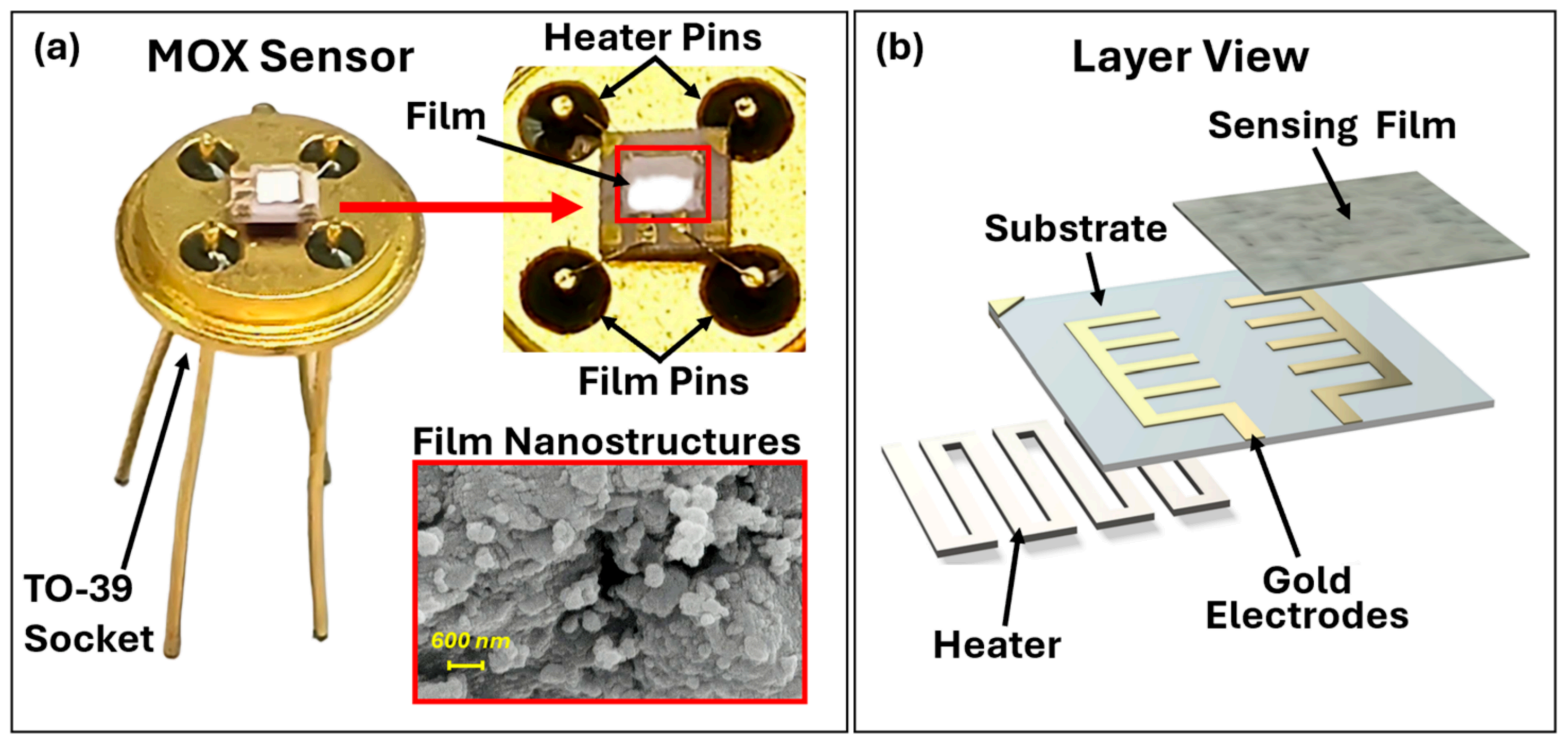
Different views of the MOX sensor device: (**a**) Assembled MOX sensor device, with the red inset showing an SEM image of a representative STN sensing film. The SEM image includes a yellow scale bar corresponding to 600 nm. (**b**) Exploded layer view illustrating the structural configuration of the MOX sensor.

Nanostructured metal oxide chemoresistive gas sensors are among the most extensively studied and widely deployed sensing devices worldwide due to their high sensitivity, low cost, simple fabrication processes, and versatility across a broad range of applications, such as environmental monitoring, industrial process control, pollution detection, public safety, and healthcare screening and diagnostics. The term nanostructured refers to the nanoscale morphology of the sensing layer, characterized by a network of interconnected nanometer-sized grains that generate a highly porous architecture (Figure 9a). This nanostructured configuration provides a high specific surface area and abundant active sites, promoting enhanced analyte–surface interactions, improved charge transport properties, and consequently optimized sensing performance [45,46,47,48]. These devices are classified as chemoresistive sensors because their operating principle is based on variations in the electrical resistance of the sensing film induced by the chemical redox reactions occurring between gaseous species in the surrounding atmosphere and the film surface. This interaction modulates the charge carrier concentration within the sensing layer, producing a measurable electrical response.

The sensing mechanism is primarily governed by surface reactions between the target gas species and oxygen ions chemisorbed on the metal oxide surface [45,49,50]. In ambient air, oxygen molecules capture electrons from the semiconductor, forming ionized oxygen species and creating a depletion layer that modifies the electrical conductivity of the material. Upon exposure to reducing or oxidizing gases, charge transfer processes alter the concentration of charge carriers, resulting in measurable resistance variations that can be correlated with gas concentration [49,50].

Chemoresistive metal oxide gas sensors are typically composed of three main parts: a substrate, a heater, and a nanostructured sensing film (Figure 9b). The substrate, made of alumina in the present work with an area of 2.53 × 2.53 mm^2^, provides mechanical support for the sensor and acts as an insulating layer between the sensing film, deposited on the top side of the substrate, and the heater, printed on the bottom side. Two interdigitated gold electrodes are patterned on the upper surface of the substrate, and the sensing film is deposited between these electrodes by means of screen-printing technology. The heater consists of a platinum meander printed on the backside of the substrate and is used to heat the sensing layer to its operating temperature, typically ranging from 300 to 600 °C.

The sensing film is a thick and porous metal oxide layer, with a thickness of 30 μm in this work, composed of an interconnected network of semiconducting nanograins, which provides a large specific surface area and numerous active sites for gas adsorption.

#### 4.1.1. Nanostructures Synthesis Through Sol–Gel Technique

The exceptional sensitivity of nanostructured metal-oxide (MOX) gas sensors arises from their high specific surface area and nanoscale grain dimensions. The large surface-to-volume ratio increases the density of active sites available for gas adsorption, while grain sizes comparable to the Debye length enable surface reactions to significantly modulate the material conductivity, producing substantial resistance changes even at low gas concentrations. Moreover, the high porosity of the sensing film facilitates gas diffusion throughout the material, allowing a larger fraction of the active layer to participate in the sensing process and thereby enhancing both sensitivity and response dynamics. To achieve these structural characteristics, the sol–gel synthesis technique was employed to fabricate highly porous networks of interconnected MOX nanoparticles with controlled morphology and grain size. The sol–gel process involves a sequence of chemical transformations that convert molecular precursors into a three-dimensional oxide network and can be summarized in the following main stages [51]:

- Hydrolysis of the precursor. Metal or silicon alkoxides are hydrolyzed in an aqueous or alcoholic solution, leading to a homogeneous solution called SOL. Some catalysts (such as acids or bases) are often added to promote hydrolysis and to obtain a colloidal suspension.
- Condensation. During the hydrolysis process, condensation reactions occur between hydroxylated species, leading to the formation of M–O–M bridges (where M is the metal and O the oxygen). This step builds an interconnected colloidal network dispersed in the liquid phase (gel). The nature of the alkoxide precursor and the solution pH are the crucial modifiable parameters to fully control particle size, degree of cross-linking, and network morphology.
- Aging. This step, typically taking from several hours to a few days, involves further polycondensation reactions within the gel network.
- Drying and calcination. The gel is first dried at low temperatures to remove water and residual solvents (typically ranging between 60 and 150 °C; however, temperatures below 100 °C are generally preferred to minimize capillary stresses and prevent film cracking). The drying step is followed by the calcination step, which is performed at significantly higher temperatures (typically 400–800 °C) to eliminate residual organic species and promote oxide crystallization. The calcination temperature and ambient humidity significantly affect the microstructure, porosity, crystallinity, and overall quality of the final MOX powder [52,53].

The sensing materials investigated in this work are ST25 and STN. ST25 consists of 75% tin oxide (SnO_2_), 25% titanium dioxide (TiO_2_), and 1% gold nanopowder; it is known in the literature that noble metal nanoparticles improve MOX gas sensor performance thanks to their catalytic behavior: they facilitate gas dissociation and surface reactions via spillover effects and modulate charge transport through the formation of metal–semiconductor interfaces. In contrast, STN is composed of 70% tin oxide (SnO_2_), 30% titanium dioxide (TiO_2_), and 1% niobium oxide (Nb_2_O_5_). The synthesis procedures, sensor reproducibility, and response repeatability in wet and dry conditions for these two materials have been previously reported and described in publications from our research group [44,54,55,56].

#### 4.1.2. MOX Gas Sensor Production and Assembly

**Figure 10:**
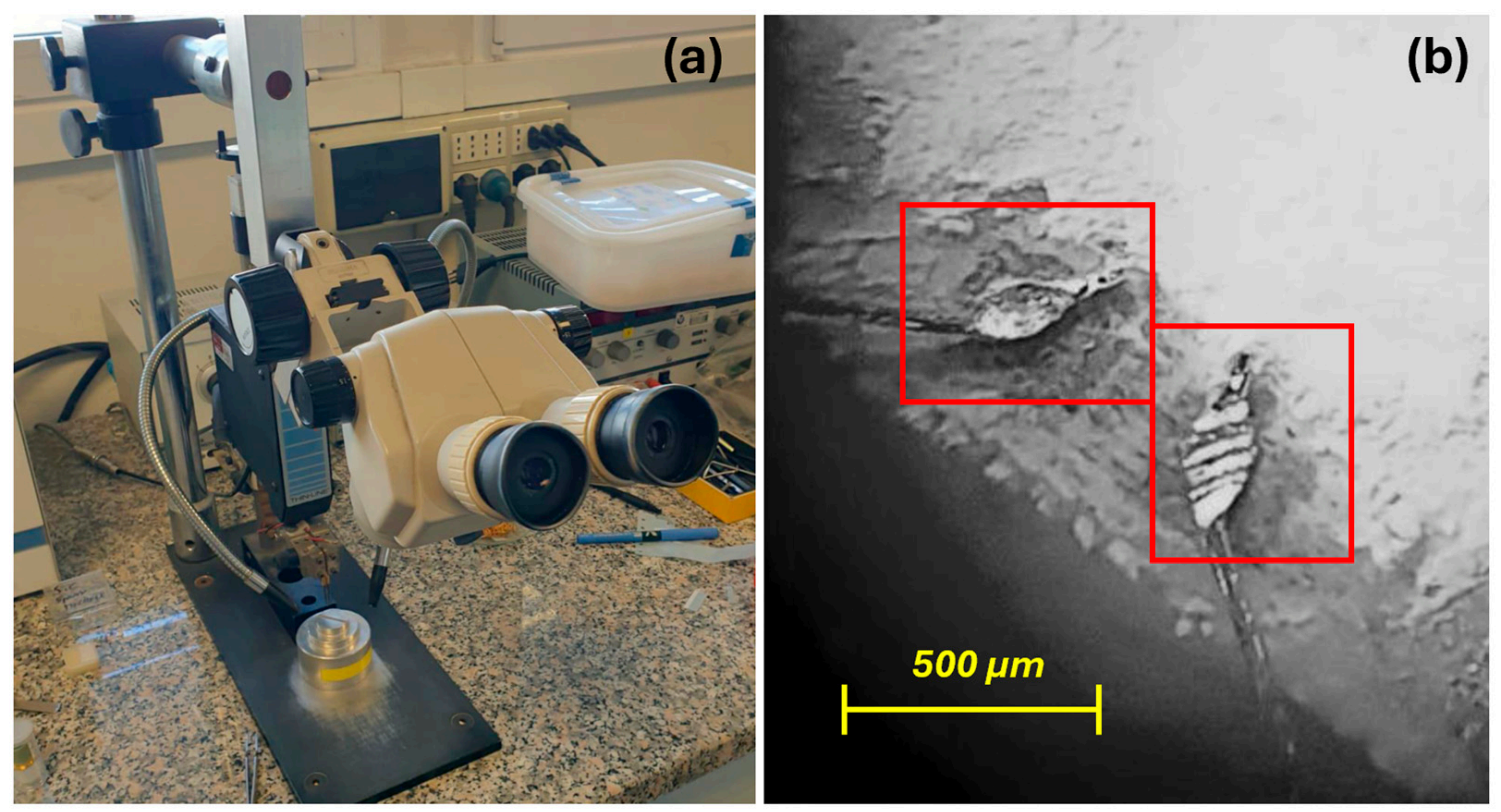
Sensor assembly and packaging process. (**a**) Thermocompression wire bonding system employed to connect the sensor to the TO-39 connector; (**b**) view of an assembled sensor highlighting the gold wire bonds on the interdigitated electrodes (red squares).

After synthesis, the nanopowder is converted into a printable, viscous paste by adding suitable organic binders. The resulting paste is then deposited onto the sensor substrate by a screen-printing machine (Aurel C920, Forlì-Cesena, Italy). The sensing film has a thickness of approximately 30 μm, which is determined by the thickness of the screen-printing mask employed during the deposition process, while the active sensing area is 1.22 × 1.6 mm^2^. Following deposition, the sensors undergo a two-step thermal treatment consisting of a drying process and a firing process, both carried out in an oven. The drying step is crucial to remove solvents and volatile organic components from the printed paste, and it was performed at 80 °C for 3 h, while the firing step (at 650 °C for 2 h) promotes the decomposition of residual organic compounds and enhances the adhesion between the sensing layer and the substrate. As the final fabrication step, the sensor is welded onto a four-pin TO-39 package and electrically connected to it through 40 μm diameter gold wires. The wire bonding process is performed by thermocompression using a bonding machine, ensuring reliable electrical connections between the sensor electrodes, the integrated heater, and the TO-39 pins (Figure 10).

### 4.2. Biopsy Collection and Preparation

**Figure 11:**
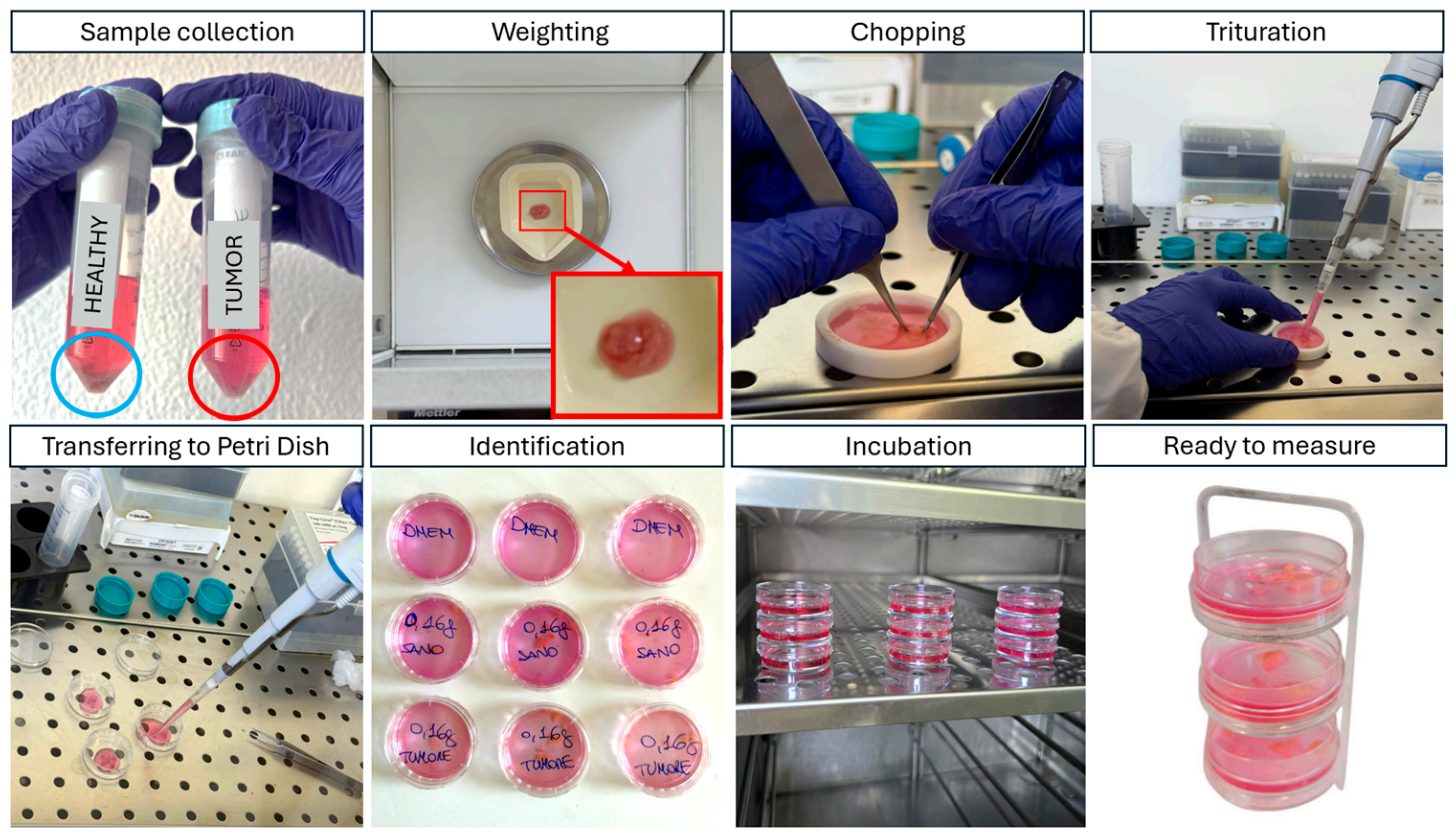
Flux diagram of the biopsy sample preparation procedure.

Two tissue samples were collected by the surgeons of the General Surgery at the Sant’Anna Hospital (Cona, Ferrara, Italy), from the resected intestinal specimen of each patient: one excised directly from the tumor mass and the other from healthy epithelial tissue, as far as possible from the tumor site. The samples were collected in two separate 50 mL tubes (Figure 11, “Sample collection”) containing DMEM with high glucose, supplemented with sodium pyruvate, 10% fetal bovine serum, 1% L-glutamine, and 1% penicillin/streptomycin (all purchased from Lonza, Milan, Italy); this complete medium is identified as DMEM throughout the paper. Then, the samples were promptly transported by means of a polystyrene foam container to ensure thermal stability throughout transit to the laboratory at the Department of Neuroscience and Rehabilitation (University of Ferrara), where all the equipment and protective systems are available to ensure that the biopsy material is prepared under aseptic and safe conditions and properly stored. The time between surgical sample collection and incubation after preparation was typically around 1 h, with an estimated variability of approximately 30 min. Initially, the tumoral and healthy tissues were weighed separately by means of a precision balance (AE 166, Mettler Toledo, Greifensee, Switzerland), in order to isolate two samples of the same weight (Figure 11, “Weighting”); any tissue excess was discarded. All samples were then washed several times with phosphate-buffered saline under a laminar flow hood (Steril-BIO BAN Compact, Angelantoni, Perugia, Italy) and chopped as much as possible into small pieces with a scalpel and forceps (Figure 11, “Chopping”). The tissue suspension was then triturated by pipetting up and down using a cut pipette tip to achieve the highest degree of tissue fragmentation (Figure 11, “Trituration”). Both fragmented tissues were subsequently equally distributed into three Petri dishes (Figure 11, “Transferring to Petri Dish”), three for healthy tissue and another three for tumor tissue, and 1.5 mL of DMEM was added to each. A further set of three Petri dishes containing only DMEM was prepared and incubated under the same conditions as the tissue samples to be used as a control/calibration sample. To ensure accurate identification, every Petri dish was labeled on its lid (Figure 11, “Identification”).

**Figure 12:**
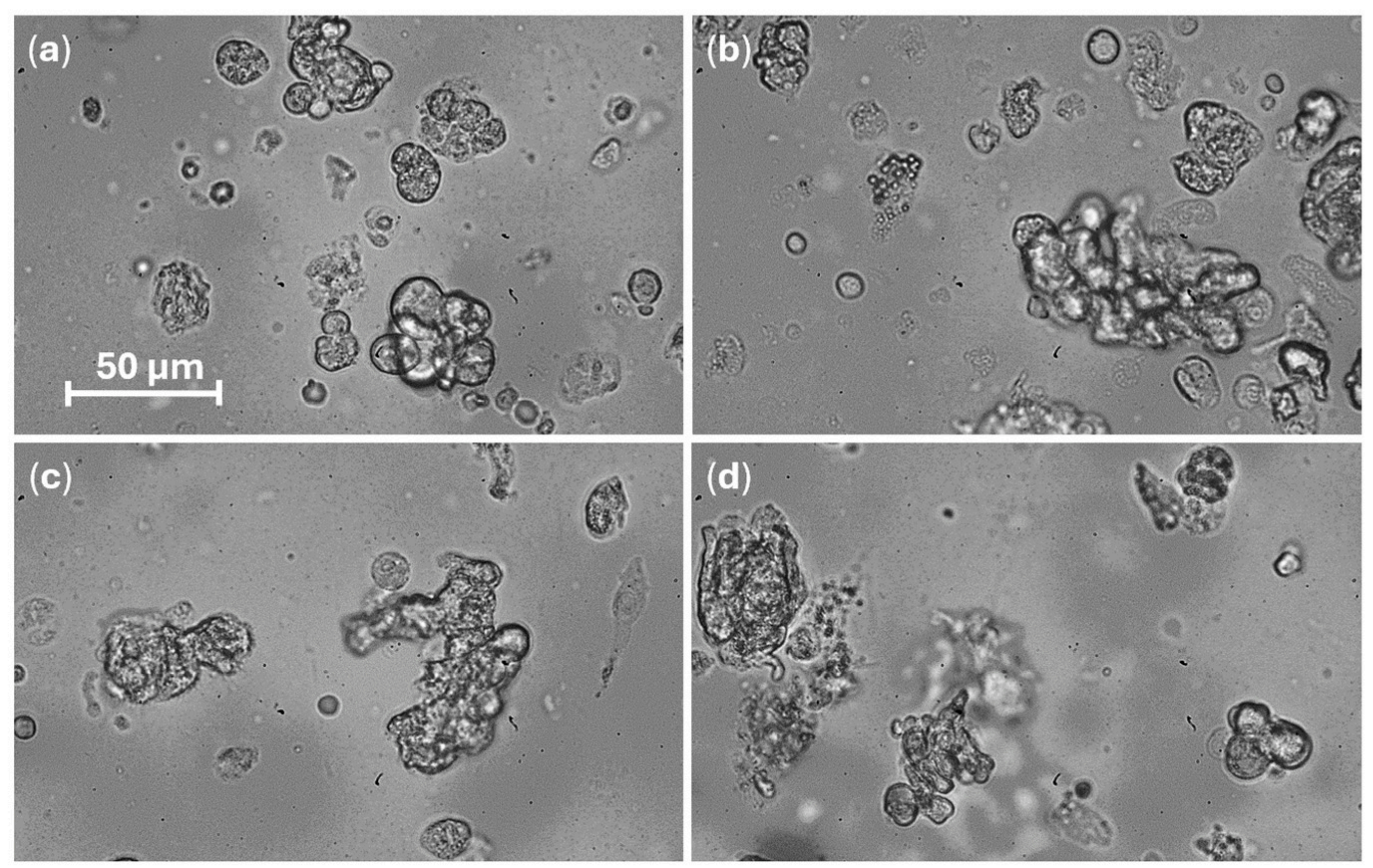
Microscopic images of dissociated biopsy tissues: (**a**,**b**) two Petri dishes containing healthy tissue; (**c**,**d**) two Petri dishes containing tumor tissue.

After labeling, the samples were placed into an incubator (Forma direct heat CO_2_ incubator, Thermo Scientific, Waltham, MA, USA) at 37 °C and 5% CO_2_ under controlled humidity (optimized parameters to allow cancer cell proliferation and growth) and were measured 2 ± 0.5 h after incubation (Figure 11, “Incubation”). The simultaneous analysis of three Petri dishes (Figure 11, “Ready to measure”) for each sample was adopted to increase the exhaling surface, thereby improving the number of gaseous compounds conveyed by the airflow to the sensor array. In other words, three Petri dishes containing healthy tissue, three containing CRC tissue, and six containing DMEM were prepared. The Petri dishes were measured simultaneously in groups of three, resulting in a single sensor measurement for the healthy tissue, a single measurement for the CRC tissue, and two measurements for the DMEM controls (one acquired first and the other last). Thus, although three Petri dishes were prepared for each tissue condition, they were measured together and did not constitute independent measurement replicates. The samples were checked after the measurements by viewing them on a screen connected to a high-sensitivity digital CMOS camera, using a 2.3-megapixel CMOS sensor (C11440-36U, Hamamatsu Photonics, Tokyo, Japan) coupled to a microscope (TE 300, Nikon, Tokyo, Japan) as shown in Figure 12.

### 4.3. Cell Culture Samples Preparation

The cell types employed in this study were Caco-2 and RKO (the American Type Culture Collection, ATCC^®^ CCL-61™, Manassas, VA, USA), cultured and maintained in DMEM.

**Figure 13:**
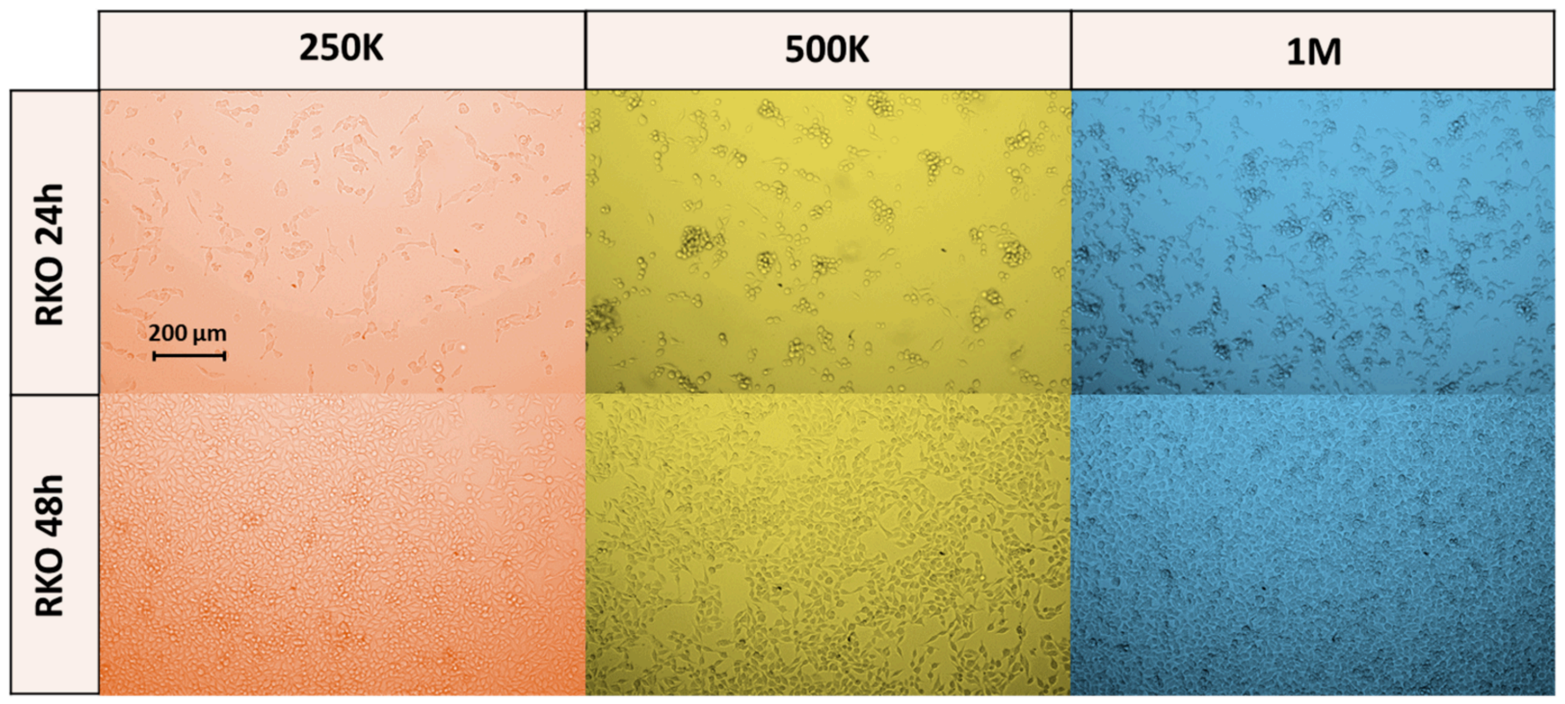
Microscopic images of representative RKO cells plated at different initial seeding densities and incubated for varying time periods. Rows correspond to increasing cell densities (250 K, orange; 500 K, yellow; and 1 M cells, blue), while columns represent incubation times of 24 h and 48 h. The scale bar represents 200 µm and is applicable to all micrographs.

To maximize the sample exhaling surface and improve the signal-to-noise ratio, three Petri dishes were measured simultaneously by stacking them in a dedicated holder (Figure 11, “Ready to measure”), replicating the same measurement protocol adopted for biopsies. Each experimental session consisted of an initial and final measurement of DMEM alone as a calibration sample, and the measurements of cell samples at different concentrations 24 and 48 h after plating, properly preserved in an incubator at 37 °C and 5% CO_2_ in a humidified atmosphere. For each sample, three independent replicate measurements were performed. In each replicate, three Petri dishes were measured simultaneously. After each measurement, the samples were checked by viewing them on a screen, connected to a high-sensitivity digital CMOS camera, using a 2.3-megapixel CMOS sensor (C11440-36U, Hamamatsu Photonics, Tokyo, Japan) coupled to a microscope (TE 300, Nikon, Tokyo, Japan); microscopic images of RKO cells are shown in Figure 13. For each cell concentration, three Petri dishes were removed from the incubator 24h after plating, measured, and subsequently discarded to avoid possible contamination; then another three Petri dishes of each cell concentration were measured after 48h. Therefore, twelve Petri dishes containing only DMEM and six Petri dishes for each selected cell concentration were prepared, corresponding to 2.5 × 10^5^, 5 × 10^5^, and 1 × 10^6^ cells per dish (3.5 cm diameter). All dishes were maintained in the same incubator.

### 4.4. Measurement Device and Data Acquisition

**Figure 14:**
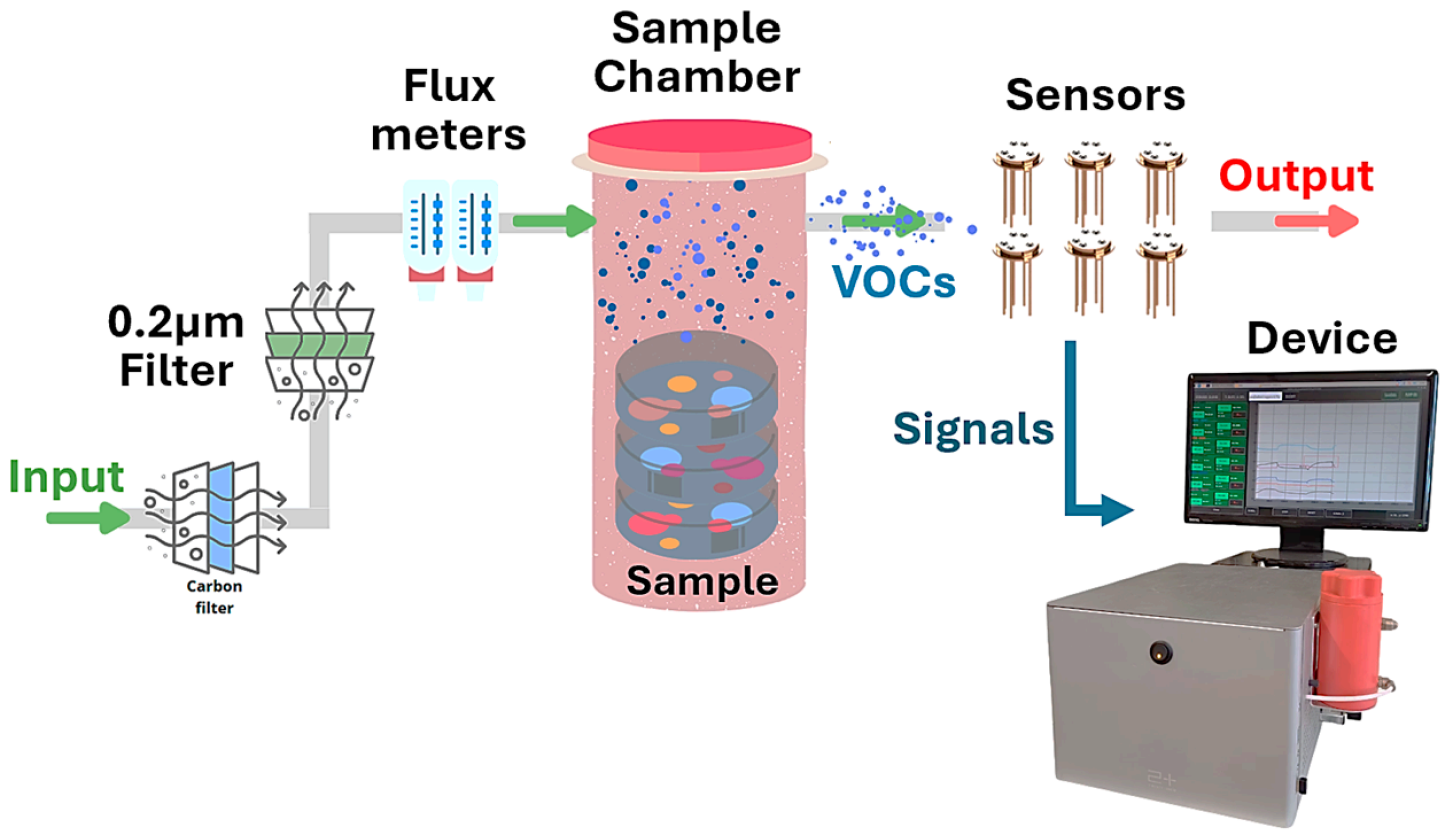
Block scheme of the device. Schematic representation of the airflow path during the analysis of volatile emissions from a sample (**left**) and photograph of the portable instrument (**right**), which integrates the entire electronic and pneumatic system for signal acquisition and analysis.

The device consisted of a portable Raspberry Pi–based prototype comprising two main subsystems: an electronic system and a pneumatic one (Figure 14).

The electronic system, currently under the patenting process, was designed to supply the sensor heater with high precision and to stably maintain it at the desired working temperature (WT ± 3 °C). This system was capable of setting the heater up to a maximum temperature of approximately 800 °C. Signal digitization and acquisition were performed through a dedicated circuit based on an operational amplifier in an inverting configuration, which converted variations in the sensing film resistance into corresponding voltage variations. These voltage signals were then digitized and acquired using a 16-bit analog-to-digital converter (ADC).

The pneumatic system performed several essential functions in order to carry the gaseous compounds toward the sensors. Ambient air was aspirated by a micropump and passed through a carbon filter and a 0.2 μm filter to stabilize humidity levels and remove most contaminants from the airflow. A sample container was fixed to the side of the device, and it consisted of a cylindrical cavity that could be hermetically sealed using a cap fitted with an O-ring, into which the sample was inserted. The sample chamber was equipped with two connectors (inlet and outlet) for the airflow. This system included a solenoid valve that could exclude the sample chamber from the airflow path (baseline state), in which the filtered air was directed straight to the sensor core to stabilize the sensors in a reference atmosphere or to reach baseline recovery after exposure to a stimulus, or include the sample chamber in the airflow path (measurement state), thereby carrying the volatile compounds present in the sample chamber headspace to the sensors for analysis. The electronic and pneumatic subsystems, as well as data acquisition and real-time data visualization, were managed by dedicated software. The sensor data, together with the environmental and temporal conditions, were stored in a .txt file where the data were organized into columns.

To minimize potential experimental biases, the measurement order of the samples was randomized. Although operators were not blinded to the tumor/healthy status of the samples, all measurements were performed according to a strictly standardized protocol. The sample holder design ensured a fixed and reproducible positioning of the sample holder inside the sensing chamber, preventing variability associated with sample placement. Temperature, relative humidity, and sensor baseline drift were continuously monitored during each measurement session and were found to have a negligible impact within individual experimental days.

### 4.5. Data Processing

**Figure 15:**
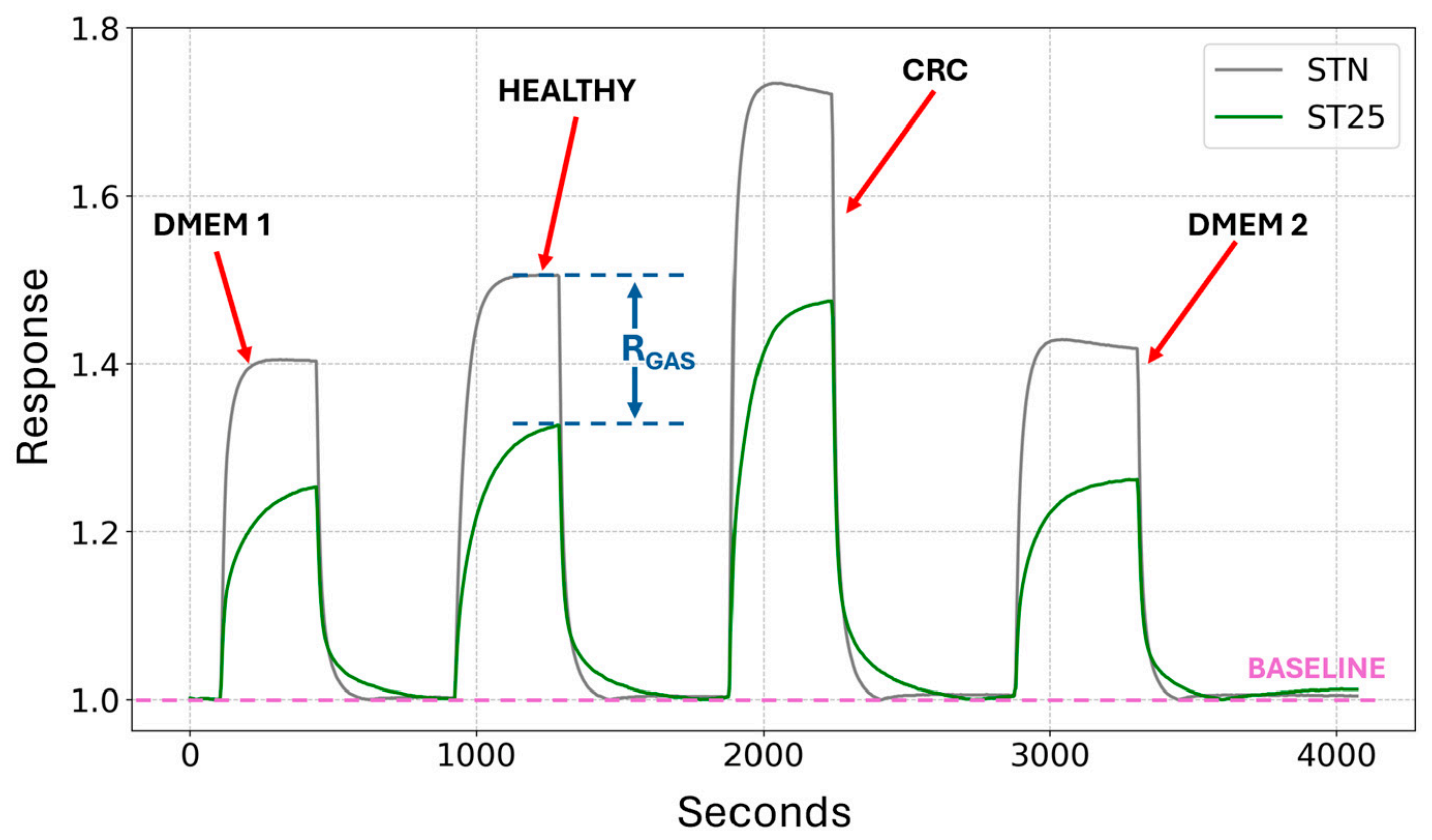
Representative sensor response plot for a single measurement day. The green and gray lines represent the response curves of the ST25 and STN sensors, respectively. The baseline (purple dashed line) was fully recovered before each measurement. In this representative measurement sequence, the response time was approximately 8 min, while the recovery time was approximately 13–14 min.

Once the .txt file containing the raw data has been obtained, the sensor signals are converted into the sensor response R in order to obtain a value completely independent of the physical quantity measured (Figure 15), by using Equation (1):

(1) $$R=\frac{V_{G}}{V_{B}}$$

where $V_{G}$ and $V_{B}$ are the sensor signals (voltage) in gas presence and in the baseline condition, respectively.

The average sensor response time was approximately 12 ± 5 min, while the average recovery time was 20 ± 7 min. In the representative example shown in Figure 15, the response and recovery times were 8 min and 13–14 min, respectively. It should be noted that both response and recovery times may vary considerably, as the analyzed samples are biological specimens that emit complex mixtures of VOCs with variable chemical composition and concentration. In contrast, the baseline drift within a measurement session was found to be negligible, as illustrated in Figure 15, with the sensors consistently exhibiting a complete and stable recovery to their baseline signal after each measurement.

Finally, the data were stored and processed in an Excel file, and all statistical and numerical analyses were performed using the Python (3.14) libraries NumPy (2.5), Pandas (3.0), Scikit-learn (1.9), Seaborn (0.13.2), and SciPy (1.18), which directly retrieved the required data from the Excel file.

### 4.6. Patient Cohort and Exclusion Criteria

A cohort of 32 randomly selected patients diagnosed with and surgically treated for CRC was included in this study, and all enrolled patients provided written informed consent for the use of their biological material for research purposes. A total of 32 matched healthy/CRC tissue pairs were initially collected; six pairs were excluded before sensor measurements due to bacterial contamination detected by microscopic inspection, resulting in 26 matched pairs included in the final analysis. Table 6 presents the available characteristics of the patient cohort involved in this feasibility study.

Only adult patients who voluntarily agreed to participate in the study and provided written informed consent were included.

Patients were excluded if they were pregnant or had previously undergone extensive neoadjuvant treatments, such as major surgery, chemotherapy, and/or radiotherapy. All participants met the predefined eligibility criteria, and the study was conducted in accordance with the principles of the Declaration of Helsinki and approved by the relevant Ethics Committee. All patients underwent surgical treatment for histologically confirmed colorectal cancer (CRC) at Sant’Anna Hospital, Ferrara, Italy.

## 5. Conclusions

This feasibility study, using ex vivo biopsy-derived samples and cell line models (RKO and Caco-2), aimed to investigate the ability of two chemoresistive gas sensors, ST25 and STN, to distinguish the odor profiles of CRC and healthy cells. The two sensors, based on nanostructured metal oxides of tin, titanium, and niobium, demonstrated a good ability to distinguish between healthy and diseased biopsies: the difference in mean responses to CRC and healthy samples was statistically significant, as assessed using a paired *t*-test (*p* = 0.00018). The effect size, evaluated using Cohen’s d, was 1.22, indicating a large effect. The data were further analyzed using LDA, which achieved good sample classification with a mean accuracy of 0.73, and ROC curve analysis on LD1-projected data, resulting in an AUC of 0.83. These sensors demonstrated an even greater capacity to distinguish between the volatile emissions of RKO cells (aggressive tumor cells) and Caco-2 cells (low-grade tumor cells), with markedly higher sensor responses to RKO emissions compared to Caco-2 cells, exhibiting a clear concentration-dependent trend. Although this is a feasibility study, innovative techniques based on these sensing technologies could be excellent future candidates for advancing and improving the detection of cancer, provided that translation to non-invasive clinical screening undergoes future validation in clinically accessible samples (e.g., breath, stool, urine, blood, etc.) and prospective validation in independent patient cohorts.

## 6. Patents

Patent Pending N. 102025000026479.

## Figures and Tables

**Table 1:** Average sensor responses to DMEM, healthy (H) and tumor (T) samples.

Sample	Average ^1^	Std. Err. ^1^	IC − ^1^	IC + ^1^
DMEM (ST25)	1.34	0.04	1.30	1.39
DMEM (STN)	1.50	0.06	1.43	1.58
H (ST25)	1.45	0.04	1.40	1.51
H (STN)	1.61	0.06	1.54	1.68
T (ST25)	1.60	0.07	1.51	1.69
T (STN)	1.72	0.08	1.62	1.82

^1^ The values presented in the table were estimated using a bootstrap statistical procedure.

**Table 2:** Mean sensor responses to healthy (H) and tumor (T) samples, normalized with respect to the corresponding DMEM samples.

Sample	Average % ^1^	Std. Err. % ^1^	IC − % ^1^	IC + % ^1^
H/DMEM (ST25)	8.5	1.7	6.3	10.7
H/DMEM (STN)	7.6	1.9	5.2	10.0
T/DMEM (ST25)	19.7	5.3	13.3	26.7
T/DMEM (STN)	15.1	4.4	9.9	21.1

^1^ The values presented in the table were estimated using a bootstrap statistical procedure.

**Table 3:** Classification report.

Class	N. Data	Recall	Precision	F1-Score
H	26	0.85	0.73	0.79
T	26	0.69	0.82	0.75
Accuracy		0.77		
Ave. Accuracy (cross-validated)		0.73		

**Table 4:** Confusion Matrix.

Class	H	T
**H**	22	4
**T**	8	18

**Table 5:** Statistical summary of clusters identified by hierarchical clustering.

Cluster	Sample Number	Sample Number (%)	Silhouette Score
C1	9	25%	0.7
C2	6	17%	0.82
C3	18	50%	0.82
C4	3	8%	0.68

**Table 6:** Patient cohort demographic information.

Feature	Abs. Value	% Value
N. patients/N. samples	26/52	50% CRC, 50% Healthy
Age, years (mean ± SD)	77.3 ± 12.4	/
BMI (mean ± SD)	25.4 ± 5.8	/
**Sex**		
Male	14	53.8%
Female	12	46.2%
**Tumor Location**		
Colon	22	84.6%
Rectum	4	15.4%
**Tumor Stage**		
I	3	11.5%
II	11	42.4%
II	9	34.6%
IV	3	11.5%
**Grading**		
Low	19	73%
High	7	27%

## Data Availability

The datasets generated and/or analyzed during the current study are not publicly available because of ethical and privacy considerations.
